# Advancing MRI with magnetic nanoparticles: a comprehensive review of translational research and clinical trials

**DOI:** 10.1039/d3na01064c

**Published:** 2024-04-02

**Authors:** Radu Lapusan, Raluca Borlan, Monica Focsan

**Affiliations:** a Biomolecular Physics Department, Faculty of Physics, Babes-Bolyai University Cluj-Napoca Romania; b Nanobiophotonics and Laser Microspectroscopy Centre, Interdisciplinary Research Institute on Bio-Nano-Sciences, Babes-Bolyai University Cluj-Napoca Romania radu.lapusan@ubbcluj.ro raluca.borlan@ubbcluj.ro monica.iosin@ubbcluj.ro

## Abstract

The nexus of advanced technology and medical therapeutics has ushered in a transformative epoch in contemporary medicine. Within this arena, Magnetic Resonance Imaging (MRI) emerges as a paramount tool, intertwining the advancements of technology with the art of healing. MRI's pivotal role is evident in its broad applicability, spanning from neurological diseases, soft-tissue and tumour characterization, to many more applications. Though already foundational, aspirations remain to further enhance MRI's capabilities. A significant avenue under exploration is the incorporation of innovative nanotechnological contrast agents. Forefront among these are Superparamagnetic Iron Oxide Nanoparticles (SPIONs), recognized for their adaptability and safety profile. SPION's intrinsic malleability allows them to be tailored for improved biocompatibility, while their functionality is further broadened when equipped with specific targeting molecules. Yet, the path to optimization is not devoid of challenges, from renal clearance concerns to potential side effects stemming from iron overload. This review endeavors to map the intricate journey of SPIONs as MRI contrast agents, offering a chronological perspective of their evolution and deployment. We provide an in-depth current outline of the most representative and impactful pre-clinical and clinical studies centered on the integration of SPIONs in MRI, tracing their trajectory from foundational research to contemporary applications.

## Abstract 2

The fusion of technology and medicine, embodied by Magnetic Resonance Imaging (MRI), has transformed healthcare. Our review highlights the novel integration of SPIONs, tracing their journey from research to advanced clinical applications.

## Graphical abstract

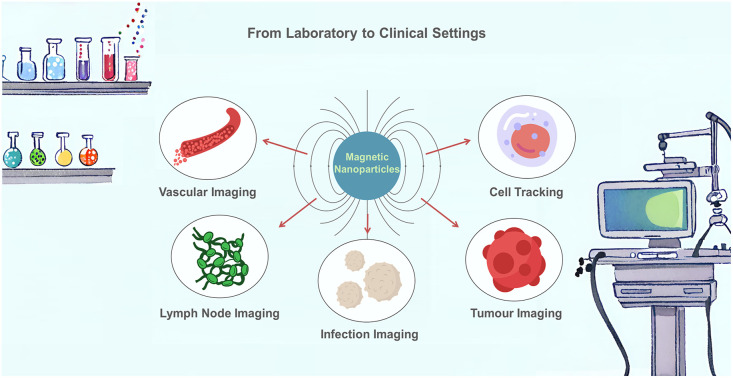

## 1. Introduction

In contemporary medicine, we find ourselves at the intersection of advanced technology and the art of healing. The profound progress in high-tech instrumentation has elevated medical imaging to an indispensable cornerstone of disease diagnosis and treatment. In the realm of medical diagnostics, we harness a plethora of multimodal imaging techniques that delve deep into the human body, providing a comprehensive view of both normal and aberrant anatomy and physiology. Over recent years, the field of medical imaging has undergone remarkable transformation, with each modality offering a unique set of advantages and limitations. Our toolkit now includes a diverse array of techniques, ranging from X-ray and Computed Tomography (CT) scans to Positron Emission Tomography (PET), Single-Photon Emission Computed Tomography (SPECT), digital mammography, diagnostic sonography and MRI.^1^

In the sphere of advanced medical imaging techniques, we find versatile tools serving a broad spectrum of diagnostic purposes, ranging from the detection and evaluation of myocardial diseases, diverse cancer types, neurological disorders, abdominal conditions, among other critical medical conditions. Each imaging modality is finely tuned for a specific application, delivering a precise function in the diagnostic process.^2^

MRI distinguishes itself through its utilization of nonionizing radiation, rendering it the preferred choice in many clinical scenarios over CT. As a noninvasive imaging technique, MRI plays a pivotal role in visualizing the human body's anatomy and physiology, applicable to both health and disease contexts. Notably, it excels in providing intricate contrast for soft tissues, enabling the differentiation between white and gray matter in the brain, making it especially valuable for diagnosing neurological disorders. Moreover, MRI serves as a vital tool in detecting various medical conditions, including ligament and tendon injuries, muscle degeneration, bone tumors, and vascular obstructions.^1,2^

However, it is imperative to acknowledge that despite these advancements, there is substantial room for further enhancement. While our current array of imaging techniques has revolutionized healthcare, opportunities for improvement persist. The integration of novel contrast agents based on nanotechnology presents significant potential, offering avenues to augment medical practice. The exploration of these state-of-the-art solutions has the capacity to reshape the landscape of medicine, potentially ushering in an era marked by even more precise diagnosis and treatment. The journey ahead involves navigating the interface of technology and healthcare, with the ongoing pursuit of a future in which healthcare is boundless in its capabilities.

## 2. MRI: principles, functionality and paths to advancement

This painless and noninvasive method is distinguished by its exceptional spatial resolution and the use of nonionizing radiation, solidifying its status as a cornerstone in the analysis of soft tissues. MRI's wide-reaching impact extends as a widely utilized biomedical method for obtaining images of the body's water-containing soft tissues. This is achieved by applying an external magnetic field to the sample, forcing the water proton's magnetic poles to line up with the field. Then, a brief radiofrequency (RF) pulse is applied at a frequency that is the same as the hydrogen atom's resonance frequency. When the RF pulse is finished, the protons release an RF signal which the MRI equipment detects and uses to create images. The short delay between RF pulses allows protons to relax and realign with the external magnetic field before changing the orientation again with the next RF pulse. The tissue's relaxation time is the duration needed by the protons (primarily those of water) to return to the initial configuration.

There are typically two main types of relaxation times: longitudinal relaxation time, or *T*_1_, and transverse relaxation time, or *T*_2_.^3^ In MRI sequences, tissue and fluid shades are described using intensity terms, which correlate with their relaxation times. High signal intensity, appearing as white on the grayscale used for image formation, often corresponds to shorter relaxation times, such as *T*_1_ relaxation. Conversely, low signal intensity, appearing as black, typically relates to longer relaxation times, such as *T*_2_ relaxation. Intermediate signal intensity, depicted as grey, usually indicates a balance between *T*_1_ and *T*_2_ relaxation times. These intensity levels are represented on a grayscale, with brighter areas indicating higher signal intensity and darker areas indicating lower signal intensity in the resulting MRI images.

*T*_1_, also known as spin–lattice relaxation, describes the process by which the net magnetization vector returns to its equilibrium value along the direction of the external magnetic field. It's often called longitudinal relaxation because it occurs along the direction of the external magnetic field. Tissues with a short *T*_1_ relaxation time will appear bright on *T*_1_-weighted images, images used for visualizing anatomy and assessing lesions, such as in the brain where gray matter appears bright and white matter appears dark. On the other hand, *T*_2_, or spin–spin relaxation, refers to the process by which the net magnetization vector in the plane perpendicular to the external magnetic field returns to equilibrium. It's called transverse relaxation because it occurs in a direction transverse to the external magnetic field and tissues with a long *T*_2_ relaxation time will appear bright on *T*_2_-weighted images. These images are valuable for detecting pathology, such as inflammation or edema, where affected areas often appear bright. Both *T*_1_ and *T*_2_ relaxation times are fundamental concepts in MRI and have important implications for image contrast. They are specific to each type of bodily tissue and largely depend on their water content, allowing for differentiation between various tissues.^4^

To further enhance MRI's imaging abilities, specialized contrast agents have been developed to alter these relaxation times. Gadolinium-based contrast agents, the currently preferred *T*_1_ contrast agents for MRI, act by shortening the *T*_1_ relaxation time of the water protons neighboring gadolinium (Gd) complexes, which leads to a brighter signal on the *T*_1_-weighted imaging sequence, therefore exhibiting positive contrast capabilities. Unfortunately, Gd in its pure form is very toxic to humans; it can lead to nephrogenic systemic fibrosis in patients with poor renal function.^3^ Gd has been shown to accumulate dose-dependently in the brain independent of renal function, as well as in other tissues (bone, liver, spleen, skin and kidney), but a link between this Gd deposition disease and clinical manifestations have yet to be demonstrated.^5^ Thus, it is becoming increasingly apparent that the current contrast agents, including gadolinium-based ones, have their limitations, especially concerning toxicity and their distribution within the body. This has prompted a quest for alternative contrast agents that could address these issues. While inorganic nanoparticles (NPs) like Gd oxide, Gd fluoride, and Gd phosphate are increasingly being explored as alternatives due to their small size and magnetic properties, they also come with their own set of limitations.

Consequently, researchers have expanded their focus to explore a broader spectrum of inorganic NPs, each with its unique characteristics. This exploration aims to identify alternative agents that can effectively overcome the limitations associated with traditional contrast agents. The field of inorganic NPs-based MRI has witnessed significant advancements, including the utilization of targeted Gd_2_O_3_ NPs, the creation of dual-function *T*_1_–*T*_2_ MRI probes, the development of hybrid MRI/fluorescent probes, and the introduction of theranostic agents specifically designed for tumor imaging. These innovations have showcased the remarkable potential of inorganic NPs in enhancing the precision and efficacy of MRI diagnostics. Notably, a study found PEGylated-Gd_2_O_3_ NPs to offer longer blood half-life, enhanced MRI contrast, and reduced toxicity compared to the commercial Magnevist.^6^ Recent advancements include the synthesis and characterization of dual contrast agents based on polydopamine NPs (PDA-NPs) targeted with hyaluronic acid (HA) and chelated with Gd^3+^. The PDA/HA/Gd^3+^ NPs demonstrated low cytotoxicity, enhanced stability in water, and targeted binding to CD44^+^ cancer cells. In imaging tests, they showed comparable or superior contrast capabilities to commercial contrast agents such as Dotarem and Barium sulfate, with higher relaxivity in MRI.^7^ These demonstrate the tangible benefits that nanotechnology can bring to the field of MRI contrast agents.

In conclusion, while current contrast agents, including various Gd-based NPs, have played a vital role in advancing MRI technology, the exploration of nanotechnology and a wider array of inorganic NPs offers a promising avenue for improvement. These advancements hold the potential to overcome the limitations associated with traditional agents, potentially revolutionizing the field of medical imaging and offering new possibilities for enhanced diagnostics and therapies.

## 3. Magnetic nanoparticles in MRI: current insights and the pursuit of enhancement

**Fig. 1:**
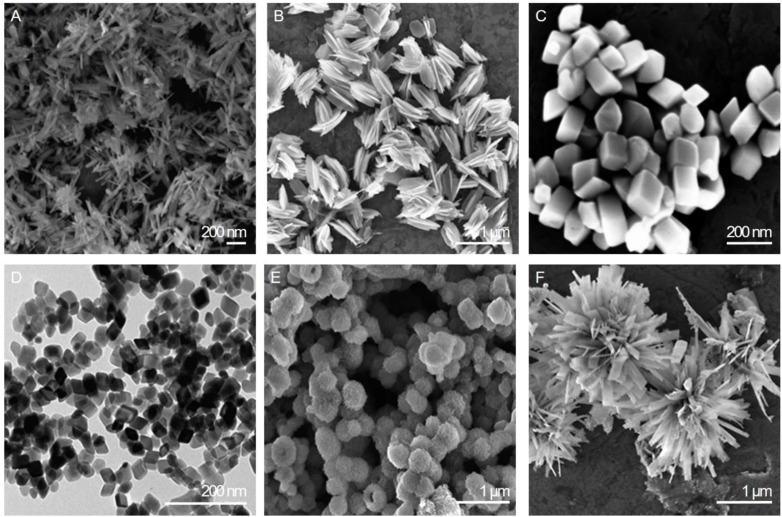
Electron microscopy images of six distinct shapes of iron oxides: (A) nanorods, (B) nanohusks, (C) distorted cubes, (D) nanocubes, (E) porous spheres, and (F) self-oriented flowers. Modified with permission from Sayed, F., Polshettiwar, V. (2014)^9^ Creative Commons – Attribution 4.0 International – CC BY 4.0 (https://creativecommons.org/licenses/by/4.0/).

With these challenges and innovations in mind, the following section delves into the promising frontier of magnetic NPs as contrast agents for MRI, exploring their potential to overcome existing limitations and transform the field of medical imaging. Precisely, iron oxide particles have emerged in the last 30 years as a safe and highly customizable alternative to Gd-based contrast agents. They have an iron oxide core, with variations such as magnetite (Fe_3_O_4_), maghemite, and hematite (Fe_2_O_3_) existing in different phases. Notably, hematite exhibits weak ferromagnetism. This distinction is crucial for understanding the magnetic properties of NPs. Furthermore, these NPs playing a significant role in MRI contrast enhancement, having the capability to alter both *T*_1_ and *T*_2_ relaxation times.^8^ These superparamagnetic capabilities of SPIONs are strongly influenced by the size, shape and coating of the particles (Fig. 1). Optimally we are looking for a narrow size distribution with homogenous physical and chemical properties, while alterations in shape can change the exposed crystal facets and the corresponding atomic arrangements within those facets, leading to significant effects on various properties, such as binding affinity, rate of tumor deposition or therapeutic efficacy.^9,10^ Based on the size range within which they reside, iron oxide particles can be divided into three categories: micro-sized iron oxide particles, approx. 1 μm (MPIOs); standard superparamagnetic iron oxide particles, 5–150 nm (SPIOs); and ultra-small superparamagnetic iron oxide particles, 5–50 nm (USPIOs).^11^

Moreover, SPIONs can create local field inhomogeneity in the tissue of choice, effectively reducing signal intensity in *T*_2_-weighted sequences (negative contrast) and increasing signal intensity in *T*_1_-weighted sequences (positive contrast). As experiments show, the *T*_2_ relaxation increases with an increasing core diameter, in contrast to the *T*_1_ shortening effect, which increases with a decreasing core diameter. Consequently, SPIONs with small core diameters function as signal amplifiers in *T*_1_-weighted sequences, while SPIONs with large core diameters are suitable for applications using the signal reduction in *T*_2_-weighted sequences. All commercially available SPIONs contrast agents are working as *T*_2_ contrast agents and reside in the range of 16–150 nm (hydrodynamic diameter).

Nonetheless, *T*_1_ and *T*_2_ are not the only MR sequences used in imaging SPIONs. Gradient echo sequences (GRE, FFE) are more sensitive to magnetic susceptibilities than spin echo sequences (SE), and this sensitivity is increased by decreasing the flip angle, extending the echo or repetition time and increasing spatial resolution.^12^ Other developments use susceptibility-weighted imaging (SWI) to improve tissue contrast with a positive contrast agent^13,14^ or even without a contrast agent^15,16^ using magnitude and phase information from the complex data of spatially highly resolved 3D gradient echo sequences and suitable post-processing algorithms that create susceptibility/phase gradient maps.^17^

### 3.1 The evolution of magnetic nanoparticles in clinical MRI

The first commercially available iron oxide NP solution was approved in 1996^18^ by the Food and Drug Administration (FDA), had a hydrodynamic size of 40–150 nm (Standard SPIOs), used a dextran coating and were designed for liver imaging (ferumoxide (Endorem^R^/Ferridex^R^), ferucarbotran (Resovist^R^)). One unforeseen side effect for Ferridex^R^ was a severe backache after bolus injection. Due to several side effects and lack of demand, Ferridex^R^ has been withdrawn from the market since 2011 and Resovist^R^ is currently available only in limited countries, like Japan. Other agents followed in 2011, namely ferumoxtran (Combidex^R^) used initially for prostate cancer lymph node metastasis imaging, and ferumoxytol (Feraheme^R^), designed for addressing iron deficiency anemia in patients with chronic kidney disease. These agents had a smaller core and hydrodynamic size, paving the way for the new USPIOs in the range of 20–40 nm. However, ferumoxytol proved some side effects and the FDA gave a black box warning regarding serious hypersensitivity/anaphylaxis reactions. But despite these limitations, ferumoxytol is among few SPIONs formulations being used today, both for the proposed application and also off-label as an MRI angiography agent in patients that cannot use Gd.^8^ Another notable mention are formulations used as oral gastrointestinal contrast agents, namely *ferumoxsil* (GastroMARK^R^(EU), Lumirem^R^(USA)) and *ferristene* (Abdoscan^R^). They belong to standard SPION type, are coated with insoluble materials (siloxane and polystyrene, respectively) and are marked as safe and effective. Ferumoxsil is today the only iron oxide NPs approved by the FDA for imaging purposes, namely gastrointestinal and bowel imaging.

### 3.2 Prospects for improved magnetic nanoparticles

While magnetic NPs have shown promise in medical imaging during the last years, challenges have arisen. Some formulations faced side effects, but advancements are continually being pursued to develop even more effective magnetic NPs for enhanced medical imaging applications.

Standard SPIOs (over 50 nm in diameter) have been faulted for (1) inadequate renal clearance after intravenous (i. v.) administration due to their large hydrodynamic size, which leads to accumulation in the body and generate persistent negative contrast spanning weeks or months, preventing further imaging investigations and hindering clinical management; and (2) the absorption in the body's iron pool can lead to clinical side effects from iron overload. Consequently, classes of positive contrast agents with very small hydrodynamic diameter are in clinical trials as we speak.^19^ These endeavors include exploring surface functionalization with advanced materials to enhance NP performance. Adding hydrophilic molecules on their surface balances the water molecules around SPIONs, further assisting in lowering *T*_2_ relaxation time. The smallest currently synthesizable iron oxide NP has a core diameter of 3–4 nm and a particle size of 5–7 nm with a monomer coating, namely very small SPIONs.^20–23^

For MRI applications, commonly used coatings enumerate poly(ethylene glycol) (PEG), poly(vinylalcohol) (PVA), or natural polysaccharides (dextran and modified chitosan), mostly owing to their lengthy shelf-life. SPIOs with neutral/hydrophilic surface and small size are phagocytized and opsonized slower than SPIOs with ionic/hydrophobic coating. Hence, in the case of standard SPIOs, monocytes and macrophages of the reticuloendothelial system (RES) remove them from the blood stream, granting a short blood half-life (*e.g.*, 8 minutes for ferumoxide). They mostly concentrate in the liver (80–90%), spleen and bone marrow. By comparison, USPIOs and very small SPIOs have a longer blood half-life (*e.g.*, 10–14 h for ferumoxytol) and can be used as blood pool contrast agents.^9,23^

The phagocytosis of SPIONs by RES organs is one of the targeting mechanisms that can be used to detect lesions in the liver, spleen, lymph nodes and bone marrow. Other passive targeting mechanisms encompass the enhanced permeability and retention (EPR) effect, nanoparticle-induced endothelial leakiness, using the tumor acidic microenvironment, or phagocytosis by inflammation-related macrophage. The EPR effect allows i. v. administered NPs to extravasate and concentrate in tumor tissue, with the percentage varying by tumor type. This effect is utilized by several clinically-approved drug nanoformulations, including Doxil™ and Abraxane™.^24^

In the absence of any EPR effect, SPIONs can bear a process called nanoparticle-induced endothelial leakiness, which rearranges the cytoskeleton by inducing gaps of over 10 μm between endothelial cells and occurs faster than phagocytosis.^25^ Moreover, using a surface charge switch triggered by the tumor's acidic microenvironment, SPIONs conjugated with pH-sensitive ligands can be activated to generate contrast and can even prompt the formation of singlet oxygen species. Last but not least, inflammation-related macrophages that can be associated with cancerous tissue but also with benign conditions like atherosclerotic plaques can also be targeted by the same mechanisms that target RES organs.^26^

Thus, bearing these considerations in mind, the strategic selection of characteristics encompassing size, shape, and surface material for functionalization holds the potential to empower the next generation of magnetic NPs. These new and enhanced magnetic NPs can broaden their capabilities, extending to specialized applications such as lymph node imaging, inflammation assessment, infection detection, vascular evaluation, cell tracking and precise tumor identification and localization. This underscores the exciting prospects for advanced MRI contrast agents with enhanced performance.

## 4. Next-generation magnetic nanoparticles in MRI: translating research to clinical trials

In contemporary clinical practice, the utility of SPIONs has extended far beyond their initial applications. These versatile NPs have found widespread use owing to their unique properties that enable both *T*_1_ and *T*_2_ relaxation shortening effects.

The most prominent role of SPIONs in clinical settings lies in harnessing their *T*_1_ and *T*_2_ relaxation properties. This dual functionality has opened the door to a spectrum of applications, with SPIONs demonstrating their efficacy in both 
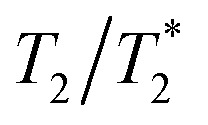
 and *T*_1_-weighted imaging; 
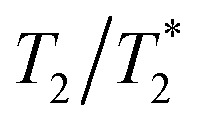
 imaging capitalizes on SPION accumulation within tissues, delivering enhanced contrast and enabling precise visualization of various anatomical structures and pathological conditions. In parallel, the *T*_1_ effect of SPIONs comes into play during their circulation within the bloodstream, further expanding their diagnostic potential.

This section delves into the extensive body of research and clinical trials that exemplify the transformative impact of SPIONs in MRI. Through a series of subsections, we explore the diverse studies and investigations that have harnessed SPIONs to advance medical imaging and patient care. From their utilization in characterizing liver lesions to monitoring lymph node metastases, SPIONs have ushered in a new era of diagnostic precision. As we navigate through these studies and trials, it becomes evident that SPIONs have become indispensable tools in the hands of clinicians and researchers. Their versatile applications and evolving formulations are paving the way for the next generation of magnetic NPs in MRI.

### 4.1 Inflammation imaging

Inflammation processes can be visualised by the high uptake of SPIONs in the macrophages. Assessing the inflammatory status of carotid plaques is an essential step in evaluating plaque instability and vulnerability to rupture and subsequent thromboembolism. Trivedi *et al.*^27^ proved in 2006 that USPIO-enhanced MRI is competent in detecting plaque inflammation *in vivo*, identifying high-risk patients. The ATHEROMA study^28^ conducted in 2009 linked an aggressive lipid-lowering therapy with an important reduction in USPIO-defined inflammation, offering value to the previous study. Further proof was presented by Morishige *et al.*^29^ one year later when the detectable signal loss in USPIO-enhanced MRI was proven to be proportionate to the degree of inflammation present, allowing the quantification of atherosclerotic plaques burden. Recent research has taken this confirmed technique to the next level. In 2019, Zheng *et al.*^30^ detected femoral artery plaque inflammation with ferumoxytol-enhanced MRI and revealed a good correlation with dynamic gadolinium-enhanced MRI. In 2022, Chan *et al.*^31^ developed a smart MRI probe using dual-targeted microparticles of iron oxide against P-selectin and VCAM-1 which allows very accurate risk stratification and is ready to be translated into clinical setting. Bonnet *et al.*^32^ made one of the first steps towards a theranostic approach to this area in 2021 when nano-emulsions of SPIONs functionalized to target galactin 3, an atherosclerotic biomarker, were loaded with alpha-tocopherol in order to reduce plaque oxidation. Recently, Segers *et al.*^33^ found that ferumoxide and ferumoxtran induce apoptosis in lipid-laden macrophages in both human and murine atherosclerosis, effect that can be avoided by using concomitant antioxidant treatment. However, this effect was not observed with ferumoxytol. These findings have significant implications for patients with advanced atherosclerosis, as they might influence disease progression, so not all results are positive.

The gold standard of imaging neuro inflammation *in vivo* is PET-CT with radiotracers in clinical setting and two-photon microscopy with fluorescent dyes in preclinical setting. While the latter has drawbacks that will not allow it to be translated into clinical practice (skull absorption of light, low penetration), the former has both its general technique disadvantages and limitations regarding acute inflammation in stroke, for example.^34^ In recent decades, USPIO-enhanced MRI have been used to track phagocytic cells in the central nervous system for various pathologies. The mechanism of enhancement different for SPIONs and for Gd. While the latter presents nonspecific inflammation data that only evaluates the integrity of the blood–brain barrier (BBB), the former is taken up directly by macrophages and can additionally identify their content and infiltration degree.^35^ Several studies in multiple sclerosis patients have proven the ability of ferumoxtran-10, when compared with Gd-enhanced MRI, to find new active lesions not detected by Gd and pinpoint lesions with aggressive behaviour, that are enhanced by both contrast agents.^36^ In the future, this dual technique should find it's way to clinical practice, although at this time the clinical studies are still in phase 1.

**Fig. 2:**
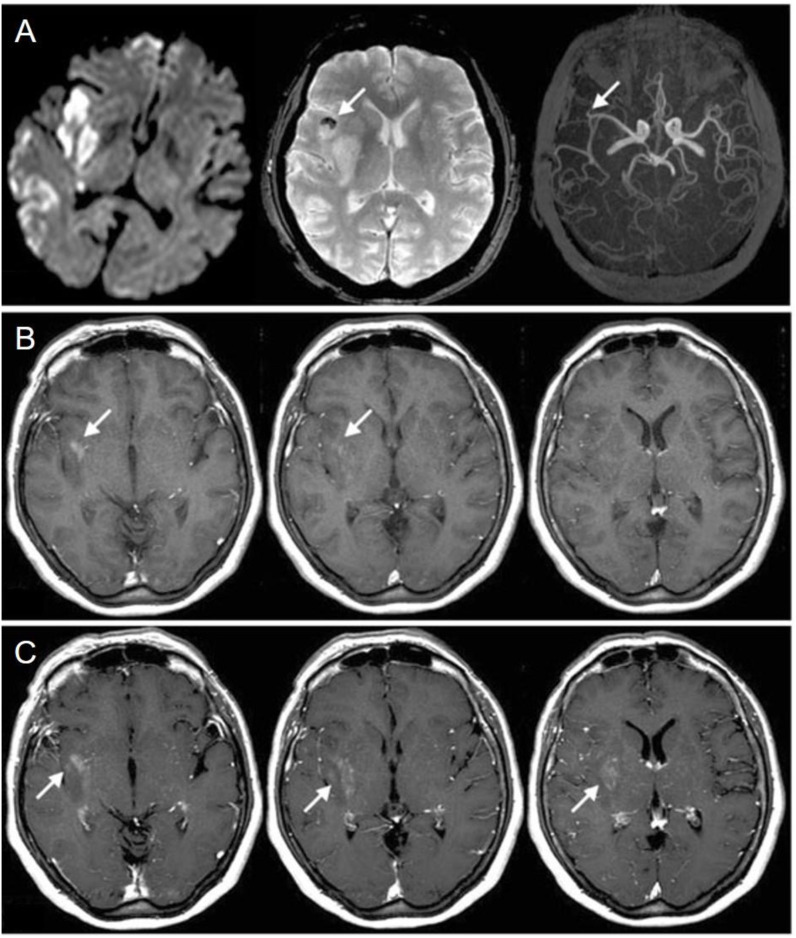
Comparison between MRI images taken on the second day before the administration of USPIO (pre-USPIO) and on the fourth day after its administration (post-USPIO) in a patient with a right middle cerebral artery infarction. (A) From (left) to (right): pre-USPIO diffusion-weighted imaging (DWI), 
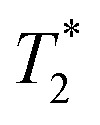
-weighted imaging (*T*_2_WI), and time-of-flight magnetic resonance angiography (TOF MRA) revealing occlusion of an insular branch (indicated by arrows); the thrombus appears hypo intense on *T*_2_WI (arrow). (B) Pre-gadolinium *T*_1_-weighted imaging (*T*_1_ WI) displaying focal enhancement within the infarct region, attributed to USPIO (arrows). (C) Post-gadolinium *T*_1_ WI showing disruption of the BBB extending beyond the USPIO-related enhancement (arrows). Copyright (2008), with permission from John Wiley and Sons.^39^

In stroke patients, macrophage response to brain ischemia can be noninvasively monitored, helping in targeting anti-inflammatory therapy to select cases.^37^ Furthermore, the works of Walter *et al.* in 2015^38^ and others before him^39,40^ have suggested that USPIOs can be a noninvasive method of tracking prognosis of ischemic stroke by setting apart inflamed brain regions without phagocytes that tend to remain viable in the long run. Fig. 2 contrasts MRI images before and after administering USPIO in a patient with a right middle cerebral artery infarction, showing occlusion and thrombus hypointensity before USPIO, followed by USPIO-related enhancement and blood–brain barrier disruption afterward.^39^ Ferumoxytol is also extensively studied in this sector. Hasan *et al.* tracked macrophage response in a brain aneurysm, selecting unstable lesions^41^ and showing reduced wall inflammation after aspirin treatment.^42^ Wall inflammation can also be observed for brain arteriovenous malformations^43^ and abdominal aortic aneurisms. In the latter case, it can provide morphologic assessment of thrombus organisation and can mark phagocytic leukocytes, electing high-risk aneurysms.^44^ Khan *et al.*^45^ showed that migraines without aura are not associated with macrophage-mediated inflammation, offering new pathways in understanding migraine pathophysiology.

The kidneys can also benefit from SPION-derived imaging techniques. First of all, it can provide useful morphological analysis by measuring the number and size of all the glomeruli in the entire kidney^46^ or asses the structural integrity of the basement membrane.^47^ Second of all, because inflammation is the main pathway to kidney fibrosis and end-stage kidney disease, diagnosis and quantification of said inflammation is of utmost importance, especially in the setting of current radiological or serological techniques that provide inconsistent and non-reproducible data. Kidney inflammation is also the main pathway of type I and II diabetes of progressing to kidney failure, owing to the proinflamatory cytokine production following hyperglycemia and hypertension. Current gold standard of assessing renal inflammation is kidney biopsy with histopathological study, which is an invasive technique that only scrutinizes 0.01% of kidney tissue; as a result about only 10% of patients undergo the procedure.^48^ Non-targeted SPIONs have been proven to accumulate in kidney macrophages by several studies, including Serkova *et al.* in 2010,^49^ and can be used to monitor kidney transplantation and transplant rejection.^50^ Targeted SPIONs have been proven to detect kidney inflammation in several pathologies that, if left untreated, eventually progress to end-stage kidney disease. Wu *et al.*^51^ developed in 2021 α_v_β_3_-targeted superparamagnetic Fe_3_O_4_ NPs for imaging of integrin α_v_β_3_, which is overexpressed in IgA nephropathy (the most common glomerular disease in the world). Serkova *et al.*^49^ used C3d conjugated NPs to weigh renal inflammation in a mouse model of lupus nephritis.

Inflammatory diseases of the bowel, namely Crohn disease and ulcerative colitis, can also be evaluated by taking advantage of macrophage uptake of SPIONs. Wu *et al.*^52,53^ presented in 2013 both the ability of detecting the presence of a pathologic process and also the quantitative assessment of disease activity, using Feridex *in vivo*.

Inflammation is also prompted by obesity at the level of the adipose tissue, leading to macrophage infiltration that is directly related to obesity-associated comorbidities. Luciani *et al.*^54^ showed in 2012 the ability to use USPIO-enhanced MR as an imaging biomarker^55^ for patients at risk for metabolic syndrome.

#### 4.1.1 Infection imaging

Inflammation and infection are closely related pathological processes. Following the development of macrophage tracking in inflammation processes, this technique was also adopted for infection detection.

**Fig. 3:**
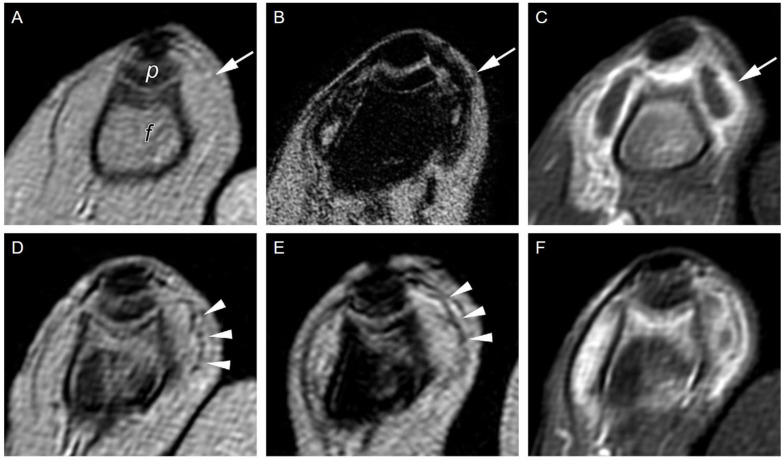
Sequential axial MR imaging tracking the progress of septic arthritis in a rabbit knee during intravenous antibiotic therapy. In the acute infection phase (A), visible synovitis with synovial thickening (arrow) is depicted on an unenhanced 
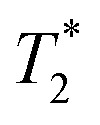
-weighted gradient-echo image, with reference to the femur (f) and patella (p). (B) Within 24 hours of USPIO injection, a pronounced signal intensity loss occurs due to iron-loaded macrophage infiltration (arrow) in the synovium, as seen on a 
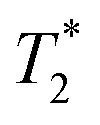
-weighted image. (C) Synovitis with signal enhancement (arrow) is observed on a gadolinium-enhanced *T*_1_-weighted image. After antibiotic therapy (D), the 
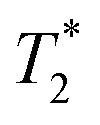
-weighted gradient-echo image reveals only focal areas of signal intensity loss in the synovium (arrowheads), which do not intensify on an image acquired 24 hours after USPIO re-administration (E). (F) Gadolinium uptake remains visible in the synovium on a gadolinium-enhanced *T*_1_-weighted image. Copyright (2011), with permission from Radiological Society of North America.^56^

SPION-based contrast agents have shown promising results in animal studies for MRI of infections. For example, studies by Lefevre *et al.*^56^ have shown a marked MR signal intensity loss in the septic knees of rabbits injected with SPIONs (Fig. 3). MR imaging tracked septic arthritis progression in a rabbit knee during antibiotic therapy, revealing synovitis and synovial thickening on unenhanced 
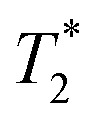
-weighted images during acute infection, followed by signal intensity loss due to iron-loaded macrophage infiltration after USPIO injection. The degree of *T*_2_-weighted signal intensity loss in SPION-treated subjects was found to correlate with the iron content in the imaged area. Furthermore, Bierry *et al.*^57^ showed that Gd alone was unable to distinguish osteomyelitis from sterile inflammation induced by mechanical damage in rabbit vertebral osteomyelitis, but SPIONs were effective for this purpose. This distinction could be made because macrophages are relatively sparse in areas of noninfectious degenerative change.^58^

In conclusion, SPION-based contrast agents show potential in MRI for detecting infections by tracking macrophages, which play a key role in both inflammation and infection processes. Additionally, SPION-enhanced MRI demonstrates significant potential in various inflammation-related applications. It aids in visualizing inflammation processes, particularly in carotid plaques, quantifying atherosclerotic plaque burden, and providing insights into plaque stability. This technique also proves valuable in neuro inflammation, enabling non-invasive detection and monitoring of conditions like multiple sclerosis and stroke, including prognosis assessment. Furthermore, SPION-enhanced MRI offers non-invasive alternatives for kidney assessment and shows promise in inflammatory bowel diseases and obesity-related inflammation. Overall, SPION-enhanced MRI expands non-invasive imaging capabilities in various medical domains, enhancing patient care and diagnosis, with ongoing research poised to advance its applications further.

### 4.2 Vascular imaging

Vascular imaging techniques will be taking advantage of the *T*_1_ shortening effects of SPIONs for the first time in this chapter. Magnetic resonance angiography (MRA) is a non-invasive medical imaging technique that uses MRI technology to visualize blood vessels and the flow of blood within the body. Not surprisingly, using contrast enhancement is a mainstay of vessel imaging, and SPION-based formulations have been comprehensively tested for this purpose in the last two decades. One of the first feasibility studies was published in 1999 when Schmitz *et al.*^59^ demonstrated good or sufficient arterial pulmonary, whole-body and lower extremity venous system visualisation in all 12 adult patients, clearly demonstrating a femoral vein thrombosis. Also in 1999, Ahlström *et al.*^60^ acquired high-quality MRA of pulmonary vasculature using Clariscan™. One of the first times magnetic NPs gained public attention as blood pool agents was in 2004, in a phase 2 study, when Prince *et al.*^61^ used ferumoxytol to reveal more aortic stent-graft endo-leaks 24 h after administration compared with CT angiography (using Gd-based products), especially detecting small and slow leaks. Ferumoxytol's coating prevents redistribution outside the vascular space, enabling delayed MRA image acquisition. This prolonged blood circulation time was further investigated in 2016 by Corwin *et al.*,^62^ who compared steady-state MRA (prolonged acquisitions that improve signal-to-noise ratio and consequently spatial resolution) with first-pass MRA, yielding a similar vessel sharpness and equivalent signal-to-noise ratio between the two.

**Fig. 4:**
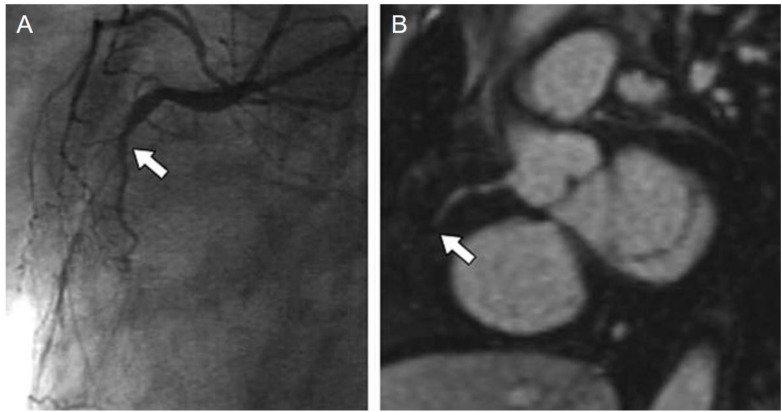
Identification of coronary artery disease through contrast-enhanced coronary MR angiography following the injection of 20 mmol Fe per kg of VSOP-C184. (A) Invasive coronary angiography confirmed the presence of occlusion in the middle part of the right coronary artery (indicated by the arrow). (B) Notably, contrast-enhanced coronary MR angiography distinctly visualizes this occlusion (indicated by the arrow). Copyright (2011), with permission from John Wiley and Sons.^66^

Several clinical phase I trials have been performed yielding promising results. In 2011, Wagner *et al.*^63^ showcased a moderate diagnostic accuracy for detecting significant coronary artery stenosis on coronary MRA using VSOP-C184 (very small superparamagnetic iron oxide particles, 184th variant of a citrate-coated preparation, batch number 050701), a USPIO formulation (Fig. 4). Identification of coronary artery disease is demonstrated through contrast-enhanced coronary MR angiography following the injection of 20 mmol Fe per kg of VSOP-C184, with occlusion confirmed by invasive coronary angiography. Although replacing CT angiography for visualisation of coronary artery disease has several advantages, including no exposure to ionizing radiation, Sakuma *et al.*^64^ presented in the same year the main drawbacks, namely long image time, lower spatial resolution, and operator dependency. Nevertheless, an ongoing phase 3 trial^65^ is currently testing coronary artery visualisation using ferumoxytol in a cohort with reduced kidney function.

The precise measurement of rCBV was taken a step further by Timms and his colleagues by developing a method called Quantitative Ultra-Short Time-to-Echo Contrast Enhanced (QUTE-CE) MRI which is well suited for measuring quantitative cerebral blood volume (qCBV), creating a 3D MRI rat brain atlas in 2017.^67^ Correlating results from awake resting-state and under isoflurane anesthesia showed significant decrease in qCBV in the neural circuitry of memory and primary senses of smell, hearing and vision (suggesting a role in consciousness) and an increase in the neural circuitry of automated functions, like preserving respiration, body temperature or cardiovascular function. Furthermore, Thrippleton *et al.*^68^ developed a protocol for evaluating cerebral small vessel disease; owing to the fact that MRI relaxometry allows USPIO uptake quantitative evaluation, they were able to measure cerebral parenchymal uptake and BBB leak.

The use of gadolinium-based contrast agents in individuals with kidney disease is a significant worry due to the potential risk of nephrogenic systemic fibrosis (NSF), a serious systemic condition first identified in 1997 in patients with kidney impairment. To prevent the occurrence of NSF in kidney disease patients, it is imperative to seek a safer alternative contrast medium that can offer comparable enhancement.^69^ For instance, individuals with kidney failure usually depend on hemodialysis to cleanse their blood, achieved through an arteriovenous fistula. Sigovan *et al.*^70^ revealed significant better performance of ferumoxytol-enhanced MRA in imaging hemodialysis fistulas as compared to non-enhanced time-of-flight MRA, greatly reducing flow artifacts. In line with the same pathology, renal graft functionality is commonly evaluated using non-enhanced ultrasound, which can yield inconclusive results. Thus, contrast-enhanced MRA proves beneficial but needs a novel contrast agent. Zamecnik *et al.*^71^ showed excellent image quality and visibility of pelvic arteries using ferumoxtran-10-enhanced 3T MRA. Timms *et al.*^72^ optimised their QUTE-CE MRI for the view of kidney vasculature, yielding great detail of renal anatomy or renal cysts and enabling quantitative morphometric analysis of abdominal and renal vessels.

Moving forward, in 2021 Shin *et al.*^73^ tested NPs with a polysaccharide supramolecular core and a shell of amorphous-like hydrous ferric oxide for the visualisation of cerebral, coronary and peripheral microvessels in rodents and of lower-extremity vessels in rabbits, showing better imaging performance than a Gd-based contrast agent, gadoterate meglumine. This is being tested in a phase 1 ongoing clinical trial^74^ as we speak.

Buch *et al.* used susceptibility-weighed MRI sequences (
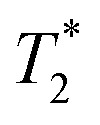
 dominant approach) to image microvasculature in several studies. In 2020^75^ they illustrated midbrain microvasculature, acquiring data at different time-points in order to avoid blooming artifacts of large vessels and detecting small vessels in the order of 50–100 μm. Next year, they were able to display vascular abnormalities and the density of small vessels in multiple sclerosis lesions, providing new insights into disease pathophysiology.^76^ In 2022,^77^ they successfully mapped hippocampal microvasculature, also quantifying tissue fractional vascular density in each of the subfields of the hippocampus; their results suggest that vascular degeneration precede tissue atrophy and are consequently able to measure atrophy and volumetric changes. This data strongly correlated both with ageing and with several neurodegenerative diseases, giving insights into disease etiology. Clinical trials of particular significance discussed in this subsection are succinctly summarized in Table 1 for reader convenience.

**Table 1:** Clinical Trial investigations for vascular imaging

Contrast agent	Dose	Condition	NCT number/approved by	Phase	Status	Ref.
Citrate-coated very small SPIONs	Cohort 1 : 1.1, cohort 2 : 2.2, cohort 3 : 2.5, cohort 4 : 3.3 (mg iron per kg of bodyweight)	Coronary artery disease	Local Ethics Committee	1, 2	Completed	63
Ferumoxytol	—	Coronary artery disease	NCT02954510	3	Ongoing	65
Ferumoxytol	Single dose, 510 mg	Reduced kidney function	Mass General Brigham Institutional Review Board	—	Completed 2018	72
Domestic polysaccharide SPIONs	—	Chronic kidney diseases	NCT05045872	1	Ongoing	74
Ferumoxtran-10	2.6 mg iron per kg of bodyweight	Reduced kidney function	Local Ethics Institutional Review Board	—	Completed	71
Ferumoxytol	4 mg per kg of bodyweight	Ischemic stroke	South East Scotland Research Ethics Committee (14/SS/1081)	—	Completed	68

SPION-based contrast agents offer exciting possibilities in vascular imaging, particularly in the context of MRA. These agents have been extensively evaluated for enhancing MRA, with early studies showcasing their effectiveness in visualizing pulmonary and venous systems, detecting arterial thrombosis, and highlighting aortic stent-graft endoleaks. The prolonged blood circulation time of some SPION formulations has been harnessed to improve MRA image acquisition. Clinical phase I trials have shown promise in coronary artery imaging, although challenges related to imaging time and spatial resolution remain. Novel SPION formulations continue to enhance vascular imaging, yielding valuable insights into various vascular pathologies and contributing to our understanding of neurodegenerative diseases.

### 4.3 Cell tracking

Over the past few years, cell and gene therapies have gained increasing popularity and have proven to be more effective, resulting in 27 approvals from FDA by December 2022. A significant challenge in gaining approval for clinical use of cell-based treatments is the complexity of assessing and pinpointing the specific impact of each mechanism that adult stem cells employ for tissue regeneration.^78^ When transplanted, mesenchymal stem cells (MSCs) can travel to the site of injury and facilitate tissue regeneration, primarily by delivering trophic and paracrine factors, for example in cerebral infarction.

MRI cell tracking emerged as a novel approach in 1993^79^ in order to examine cell survival and migration following grafting. A groundbreaking study in 2002^80^ was the first to explore cell tracking for brain injury repair, marking a significant milestone in the evaluation of treatment outcomes.

Numerous other studies over the last two decades restated SPION's reliable tracking abilities by labeling MSCs. Amsalem *et al.*^81^ tracked MSCs injected into groups of rats that had suffered a myocardial infarction, while Chapon *et al.*^82^ compared MRI imaging with PET results, confirming the homing of the NPs to the injured site for up to 6 weeks after. Lee *et al.*^83^ tracked MSCs labeled with ferumoxytol that were introduced through stereotactic injection into the hippocampi of a transgenic mouse model with familial Alzheimer's disease for 14 days, demonstrating the effectiveness of this approach. Ferumoxytol was again tested by Hamilton *et al.*^84^ in 2019 in a mouse model of osteoarthritis, where it helped showing the anti-inflammatory characteristics of labeled MSCs on synovial inflammation following intra-articular injection.

Yan *et al.*^85^ used magnetically targeted iron oxide@polydopamine-labeled human umbilical cord mesenchymal stem cells to reduce the area of infarcted cerebral tissue and facilitate microglial shift to a neuroprotective and pro-neuroinflammative phenotype. Similar particles were used by Duan *et al.*^86^ in 2020 in the treatment of femoral head osteonecrosis, optimizing tissue repair ability, while Li *et al.*^87^ used Fe_3_O_4_@polydopamine NPs as a therapeutic strategy for burn wound healing.

Furthermore, SPION seem to enhance adipogenesis and osteogenesis by their presence alone^88,89^ or in the presence of a magnetic field.^90^

Li *et al.*^91^ showed in 2019 that SPION facilitate the movement of mesenchymal stem cells toward sites of injury while having no negative effects on their properties; however, Kolecka *et al.*^92^ showed a potential negative effect on chondrogenesis, using Endorem in a canine model while successfully tracking adipose stem cells.

**Fig. 5:**
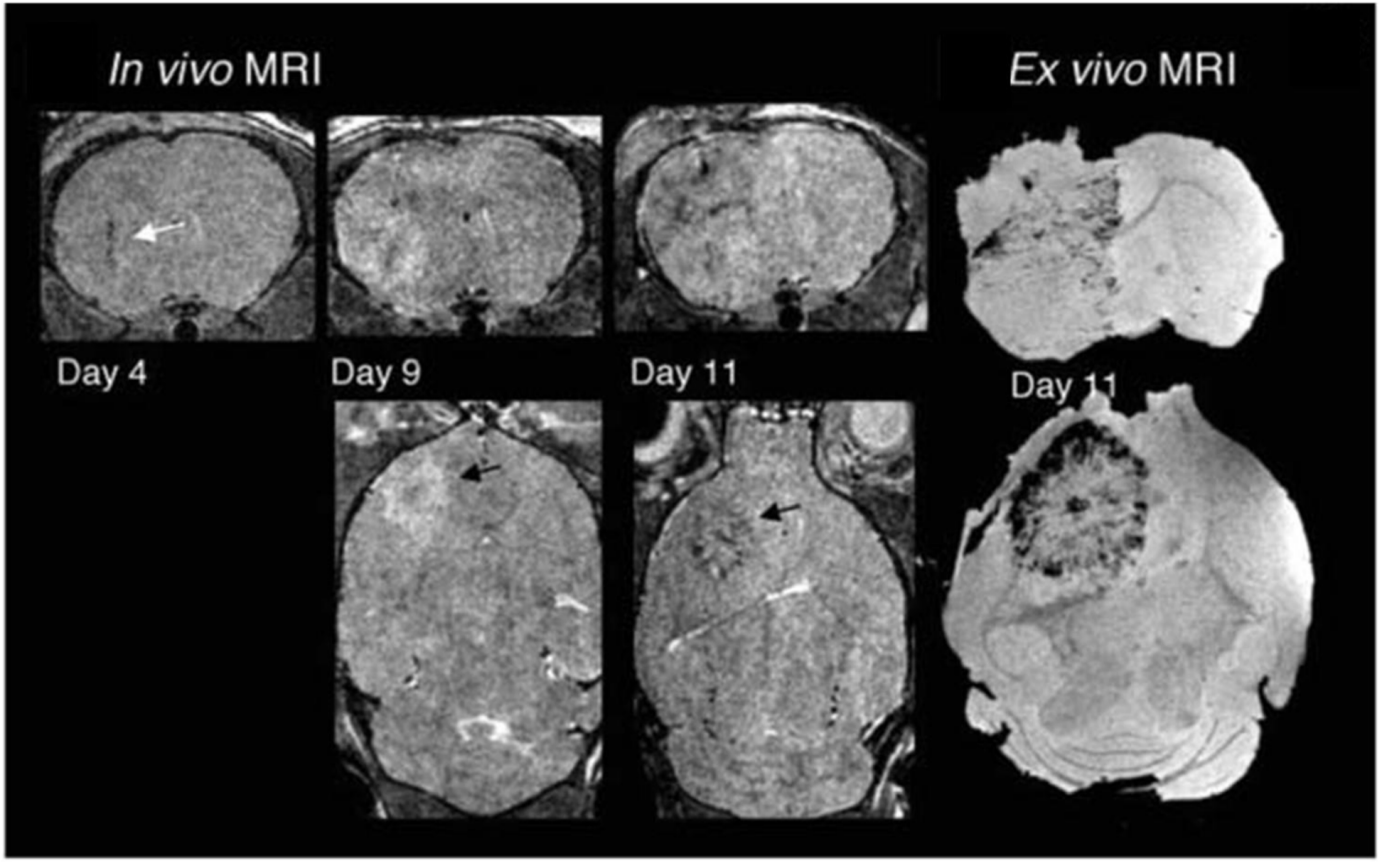
Targeting tumor vasculature, Sca-1 positive bone marrow cells, magnetically labeled, were tracked using serial MRI in tumor-bearing mice. Three-dimensional RARE images acquired on days 4, 9, and 11 revealed dark regions emerging within and around the tumors, signifying the incorporation of labeled cells into both tumor vasculature and parenchyma. By day 11, a dark rim became evident at the tumor's periphery. Corresponding *ex vivo* gradient images on day 11 confirmed MR evidence of labeled cell incorporation, indicating that neovascularization primarily occurs at the tumor periphery in later stages of tumor development. Modified with permission from Tang, C. *et al.* (2010)^93^ Creative Commons – Attribution 2.5 Generic (https://creativecommons.org/licenses/by/2.5/).

Leveraging MSCs for targeted gene therapy and NPs-assisted tracking offers innovative solutions in clinical oncology. Noninvasive MRI, aided by SPION, enhances precision in tracking therapeutic MSCs, by leveraging their intrinsic tumor-seeking properties^93^ (Fig. 5). Sca-1 positive bone marrow cells, magnetically labeled, were tracked using serial MRI in tumor-bearing mice, revealing dark regions within and around tumors on days 4, 9, and 11, indicating incorporation of labeled cells into tumor vasculature and parenchyma. By day 11, a dark rim appeared at the tumor's periphery, confirmed by *ex vivo* gradient images, suggesting neovascularization primarily occurs there in later tumor stages. Hsu *et al.*^94^ developed in 2019 a state-of-the-art theranostic technique for MRI real-time tracking of the tumor infiltration of therapeutic stem cells, namely polyethylene glycol-coated superparamagnetic iron oxide-labelled placenta-derived mesenchymal stem cells, in a mouse model of glioblastoma. Hunger *et al.* tracked T-cells by labeling them with SPION in order to monitor T-cell activation immunotherapies against glioma.^95^

SPIONs have demonstrated their reliability in tracking MSCs, supporting research in various conditions such as myocardial infarction, Alzheimer's disease, and osteoarthritis. Additionally, SPIONs show promise in promoting tissue regeneration. To fully harness the potential of stem cell therapies and ensure their effectiveness, it is imperative to develop techniques for comprehensively understanding the biodistribution and fate of administered cells. MRI cell tracking, particularly when employing SPIONs, holds substantial potential for advancing cell-based treatments and personalized medicine.

### 4.4 Lymph node imaging

Lymph node staging is paramount for choosing the right therapeutic strategy in any type of cancer and can predict patient survival time. In practice, we have imaging options that include ultrasound and non-contrast-enhanced CT or MRI and surgical options consisting of lymph node removal and histological interpretation. Imaging lymph nodes relies on the assessment of changes in size and morphology only, resulting in a low sensitivity and an even lower specificity, especially when compared to USPIO-enhanced MR imaging. PET managed to surmount some of these drawbacks but is still constrained by its resolution limit.^96^ Surgical intervention is invasive, prone to complications and only studies the perilesional area, which often leads to an underestimation of metastasis by missing far-away ones, especially in the pararenal and internal iliac region. Moreover, the two techniques cannot detect very small lymph nodes, which can be metastatic too.

**Fig. 6:**
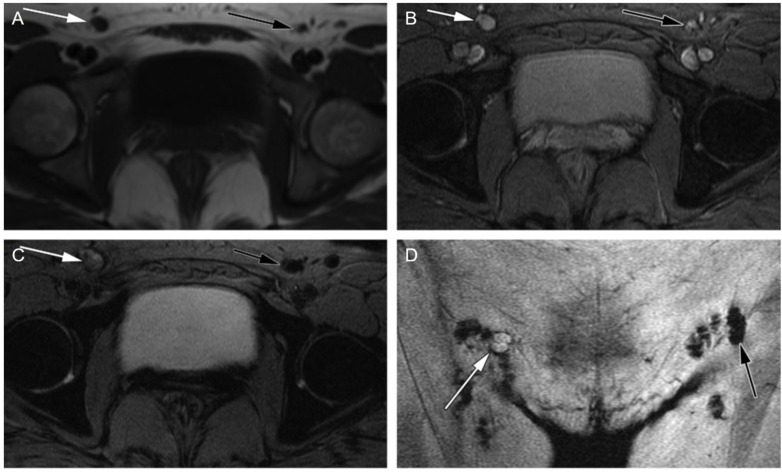
Case of a malignant inguinal node in a 47 year-old patient with vulvar cancer. The axial precontrast *T*_1_-weighted GRE image (A) reveals a borderline enlargement of the right inguinal node (white arrow) compared to the left inguinal node (black arrow). In the axial precontrast 
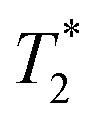
-weighted GRE image (B), both inguinal nodes (arrows) appear bright. Following contrast administration, the axial postcontrast 
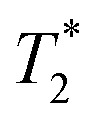
-weighted GRE image (C) demonstrates that the right inguinal node (white arrow) remains bright, while the left inguinal node becomes dark (black arrow). Additionally, the coronal post contrast 
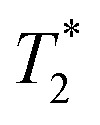
-weighted GRE image (D) provides valuable information for surgical planning, illustrating the relationship of the nodes to the inguinal fold. These imaging characteristics are indicative of a malignant right inguinal node in MR lymphography, a diagnosis that was subsequently confirmed through histologic analysis, while the left inguinal node was found to be benign. The recommended USPIO dose of 2.6 mg kg^−1^ is diluted in 100 ml of normal saline and infused over 30 minutes. Copyright (2009), with permission from Radiological Society of North America.^104^

USPIOs with a hydrodynamic diameter of 20–30 nm and a dextran coating have a long blood circulation time, making them suitable for MR lymphography. Over time, USPIOs that extravasate into tissues are collected in the lymphatic system and ingested by lymph node macrophages. This process decreases the signal intensity in *T*_2_ and 
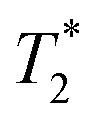
-weighted MR imaging while increasing the *T*_1_ signal. Metastatic lymph nodes, which often have a reduced number and function of macrophages, lead to lower USPIO uptake and subsequent signal modification. This can serve as an indirect method to diagnose metastatic lymph nodes, particularly those that are remote or small. Further studies showed that GRE 
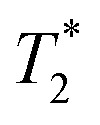
-weighted sequence showed the best nodal characterisation compared to fast spin-echo *T*_2_-weighted ones, the latter being useful for anatomic localization.^97^ This technique has been demonstrated in clinical trials for several malignancies (Fig. 6), including head and neck,^98^ breast,^99^ lung,^100^ esophagus,^101^ stomach,^102^ rectum,^103^ gynecological,^104^ bladder and prostate cancers.^105^

Ferumoxtran (Combidex® (USA), Sinerem® (EU)) and ferumoxytol (Feraheme® (USA), Rienso® (EU)) have been in clinical trials for lymph node imaging but didn't make it into practice. For example, Sinerem had its application removed by Guerbet from European Medicines Agency in 2007 in a phase 3 study due to the not definitely proven specificity of lymph node detection in pelvic carcinoma patients. Feraheme was already approved for anemia therapy as iron replacement, and was used off-label as a replacement for Combidex. Unfortunately, in 2015 FDA issued a “Boxed Warning” regarding serious risks of fatal anaphylaxis and furthermore the nodal contrast between benign and malignant was less prominent even with triple dose of feraheme, compared to Combidex.^106^

Ferumoxtran gained new-found interest in 2013 when the Radboud University Medical Center in The Netherlands started the process of producing Combidex under the same specifications as previously described, to use especially in the setting of prostate cancer.^107^ A meta-analysis by Woo *et al.* in 2018 compared the diagnostic accuracy of MRI in finding pelvic lymph node metastasis in patients with prostate and bladder cancer between 1980–2003 and 2000–2017, finding an improved sensitivity (0.39 to 0.56) and specificity (0.82 to 0.94) and attributing the effect to the use of USPIOs. Nevertheless, the sensitivity is still poor and a negative USPIO-MRI examination does not rule out the requirement of surgery.^108^ A lot of research is carried out in Nijmegen by Professor Tom Scheenen and his team, with mixed results. When comparing Gadolinium-Prostate Specific Membrane Antigen (Ga-PSMA) PET/CT with ferumoxtran-10 NP-enhanced MRI in prostate cancer lymph node assessment, both modalities identified suspicious lymph nodes that were unnoticed by the other, with the latter proving better in detecting smaller suspicious lymph nodes, suggesting a complementary role.^109^ An ongoing phase 3 trial^110^ is putting ferumoxtran-10's ability to the test against unenhanced MRI. When evaluating lymph nodes in rectal cancer, USPIO-enhanced MRI was not able to differentiate between small inflammatory *versus* metastatic nodes *in vivo* on a 3T machine, warranting improvements in order to correlate smaller lymph nodes to histopathological findings.^111^ FerroTrace and Indocyanine Green are being used in a phase 1 and 2 trial^112^ for sentinel lymph node mapping in colorectal cancer. They also developed a new reading algorithm for discerning benign from malignant lymph nodes in head and neck cancer patients.^113^ Peter Choyke^114^ and his team studied the detection of lymph nodes by ferumoxytol-enhanced MR lymphography in genitourinary malignancies, yielding promising results with a high sensitivity of 98.0% but an average specificity of 64.4%.^115^ To overcome these challenges a few methods are trying to be employed. The use of a 7T MRI system can improve the intrinsic signal-to-noise ratio and, moreover, can further decrease the 
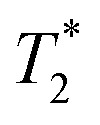
 signal after USPIO uptake because of increased susceptibility effects.^116^ Research done by He and Wei in 2016 claim diffusion-weighted sequences can have a role in discerning benign and malignant lymph nodes in cervical cancer, showing statistically significant differences in ADC values, granting a high specificity but a low sensitivity to the technique.^117^ Further research is needed in these areas.

**Fig. 7:**
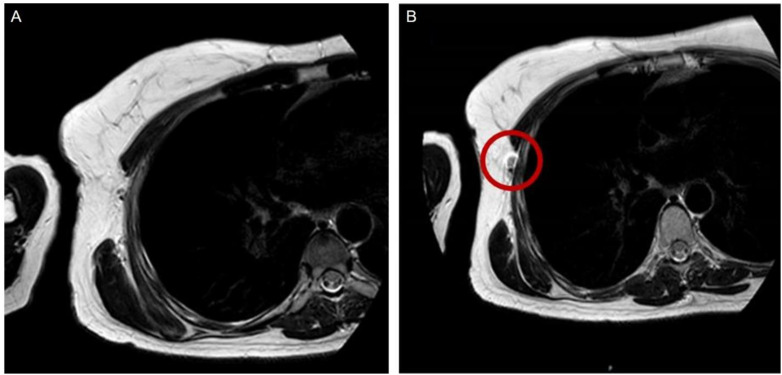
(A and B) Illustrates the visualization of the sentinel lymph node with MRI both before and after the administration of SPIO. Enhancement of the sentinel lymph node becomes apparent following the injection of SPIO, as highlighted by the red circle. Redistributed with permission from Jazrawi, A. *et al.*, (2021)^122^ Creative Commons – Attribution 4.0 International – CC BY 4.0 (https://creativecommons.org/licenses/by/4.0/).

Sentinel lymph node status is pivotal in both breast and skin cancer patients. Great strides are undertaken to find a minimally-invasive technique for accurately assessing them and sack the gold standard of axillary lymph node dissection. Motomura *et al.* have showed that sentinel lymph node status can be predicted using SPION-enhanced MRI with a low dose of ferucarbotran (Resovist) with the incorporation of a fat-suppression sequence with a sensitivity of 100% and specificity of 96%, being able to detect micro metastasis as small as 1.7 mm.^118^ They also proved equivalent accuracy of the technique on 1.5T machines.^119^ These studies were used as a background in the MAGMEN feasibility study, which correctly predicted some of the metastasis of extremity melanoma using a low-dose SPIONs injected subcutaneously.^120^ Karakatsanis *et al.* developed another technique for minimally invasive axillary mapping; in a phase 2 trial published in 2021, by integrating SPION-enhanced MR lymphography with Magnetic-guided Axillary UltraSound (MagUS) and biopsy, providing comparable results with axillary lymph node dissection^121^ (Fig. 7). The clinical trials highlighted in this subsection, which stand out in terms of relevance and representativeness, are consolidated in Table 2 for easy reference.

**Table 2:** Clinical Trial investigations for lymph node imaging

Contrast agent	Dose	Primary cancer type	NCT number/approved by	Phase	Status	Ref.
FerroTrace	—	Colorectal	NCT05092750	1, 2	Completed 2023	112
Ferucarbotran	Single dose, 6 ml	Breast	Osaka Medical Centre for Cancer and Cardiovascular Diseases Review Border	—	Completed 2016	118
MagTrace	Single dose, 2 ml	Breast	Regional Ethics Board in Uppsala (DNR 2016 – 385)	2	Completed 2021	121
Iron oxide crystalline NPs covered wtih dextran	2.6 mg iron per kg of bodyweight	Oesophageal	Medical Ethical Committee of the University Medical Center Groningen	—	Completed 2009	101
Low-molecular weight, iron oxide coated with dextran	2.6 mg iron per kg of bodyweight	Rectal	—	—	Completed 2006	103
Ferumoxytol	Cohort 1 : 4, cohort 2 : 6, cohort 3 : 7.5 (mg iron per kg of bodyweight)	Prostate	Review Board of National Institutes of Health, Bethesda	1	Completed 2012	107
Ferumoxtran-10	2.6 mg iron per kg of bodyweight	Prostate	NCT04261777	3	Ongoing	110
Ferumoxtran-10	2.6 mg iron per kg of bodyweight	Head and neck squamous cell carcinoma	NCT03817307	—	Ongoing	113 and 123
MagTrace	Cohort 1 : 5.6–14, cohort 2:2.8–3.4, cohort 3 : 0.6–1.4 (mg iron per kg of bodyweight)	Melanoma	NCT03898687	1	Completed 2020	120 and 124

In summary, lymph node staging is of utmost importance in cancer diagnosis and treatment planning. Existing methods such as ultrasound, non-contrast-enhanced CT or MRI, and surgical interventions have their limitations in terms of sensitivity and specificity. Iron oxide NPs-enhanced MR imaging has emerged as a promising tool to overcome these limitations, allowing for the detection of metastatic lymph nodes, especially small or distant ones. Clinical trials have demonstrated the potential of USPIOs like ferumoxtran and feraheme in various malignancies. However, challenges remain, and ongoing research aims to enhance the accuracy and applicability of this technique, including exploring higher magnetic field strengths, diffusion-weighted sequences, and innovative approaches for sentinel lymph node assessment. These developments signify a significant step forward in improving lymph node staging and, consequently, patient care across different cancer types.

### 4.5 Tumour imaging

Tumor detection using SPION relies on three primary mechanisms.^125^ Firstly, upon introduction into the bloodstream, SPIONs are swiftly sequestered by macrophages within the Mononuclear Phagocyte System (MPS), spanning the liver, spleen, lymph nodes, and bone marrow. Secondly, the EPR effect takes center stage in solid tumors, where the abnormal angiogenesis results in larger vessel pores (100+ nm),^126^ facilitating the infiltration of SPIONs into the tumor tissue. Lastly, active targeting strategies involve customizing SPIONs with ligands that bind to specific tumor markers, such as folate receptors, transferrin receptors, and epidermal growth factor receptors on cancer cells^127,128^. This targeted approach combines the passive EPR-based accumulation with active binding to tumor markers, although its efficacy remains a subject of ongoing debate. As thousands of recently published research papers suggest, the EPR effect is very heterogeneous in humans,^129^ varying according to tumor type, location, blood perfusion and the properties of the chemotherapeutic agents.^130^

Wang *et al.*′s study^131^ introduces a method to enhance the delivery and distribution of ultrafine SPION (under 5 nm) within tumors by fully taking advantage of the EPR effect. These ultrafine SPION can easily exit blood vessels and penetrate tumor tissue due to their small size, then cluster in the tumor's acidic environment to prevent re-entering the bloodstream. *In vivo* imaging showed initial “bright” *T*_1_ contrast in tumor blood vessels and surroundings in the first hour, shifting to “dark” *T*_2_ contrast within the tumor after 24 h, indicating cluster formation in the tumor's interstitial space.

In the liver, uptake by the Kupffer cells (the autochthonous macrophages) lets us achieve the contrast needed, owing to the fact that primary or secondary liver malignancies do not express Kupffer cells. In *T*_2_-weighted images, the presence of SPIONs in normal liver tissue results in a dark appearance, while the liver tumor remains conspicuously bright. This distinction arises from the reduced accumulation of SPION in the tumor due to its lower phagocytic activity.

**Fig. 8:**
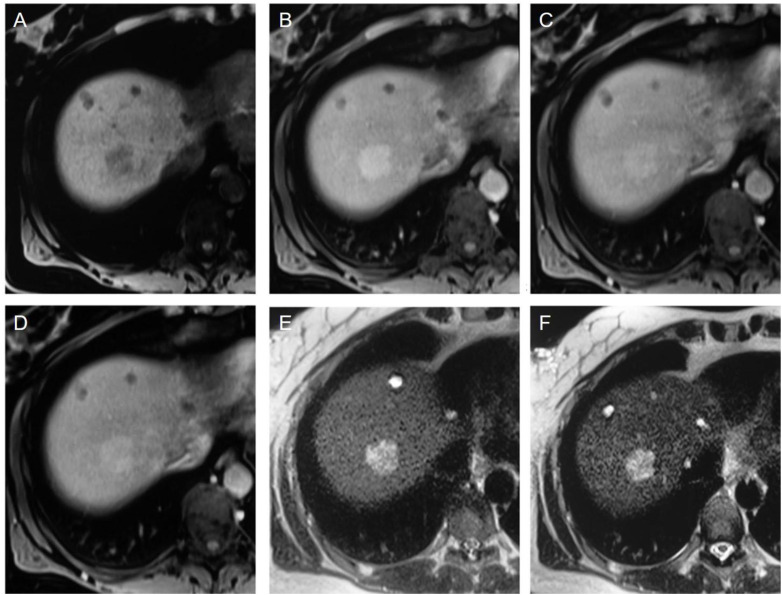
(A–F) Showcases of hepatic adenoma. In the unenhanced *T*_1_-weighted VIBE image (A), a nodule is visible, displaying heterogeneous hypointensity in comparison to the surrounding right liver lobe tissue. Following the slow bolus administration of Resovist, the nodule undergoes enhancement in the arterial phase (B) without significant wash-out during the portal venous (C) and equilibrium phases (D). On the unenhanced HASTE *T*_2_-weighted sequence (E), the lesion appears hyperintense compared to the liver parenchyma, with no significant alterations noted in the 10 minutes contrast-enhanced acquisition (F) upon visual assessment. However, quantitative analysis reveals a 33% signal loss in the nodule, which still appears hyperintense due to a 53% signal loss in the surrounding parenchyma. It's important to note that all the images (A–F) exhibit small cysts in the anterior portion of the liver parenchyma. Copyright (2009), with permission from John Wiley and Sons.^132^

Liver imaging was the application for the first NP-based iron oxide contrast agent approved by the FDA (ferumoxide) and also for the later developed ferucarbotran. They were used in clinical practice to discriminate between malignant (hepatocarcinoma and metastasis) and benign (adenoma, hemangioma, focal nodular hyperplasia) hepatic lesions^132,133^ (Fig. 8). The two compounds were compared with each other by Chen *et al.* and no significant difference was found in *T*_2_-weighted signal intensities or contrast-to-noise ratio.^134^

Meanwhile, these two contrast agents have been withdrawn from the market (except ferucarbotran in Japan), citing multiple reasons. One specific problem was the inability to differentiate normal liver from a well-differentiated hepatocellular carcinoma, which contains high amounts of macrophages. Gadolinium-based contrast agents are uptaken by hepatocytes instead of macrophages so they don't exhibit this limitation.^135^ SPIONs can still be a safe alternative for discerning liver lesions in the setting of limited kidney function (glomerular filtration rate < 30 ml min^−1^ per 1.73 m^2^) or just wishing to avoid nephropathy and the risk of nephrogenic systemic fibrosis.^69^

Similar to the liver, SPION accrue in the spleen, allowing the detection of metastasis and the rare spleen primary malignancies.^136^ Further uses encompass the detection and separation of heterotopic splenic tissue, particularly accessory spleens or splenosis. Accessory spleens are a common finding but can pose diagnostic difficulties when located intrapancreatically and need to be differentiated from pancreatic cancer. Splenosis is a benign entity, with the greatest importance being the need to distinguish them from more sinister pathology.

Contrast-enhanced MRI is commonly used to visualize brain tumors and assess BBB integrity. However, when compared to Gd-based agents, ferumoxytol did not demonstrate superiority in detecting brain cancer lesions with contrast-enhanced MRI.^137^ The advantage of SPIONs and USPIOs over small molecule Gd chelates lies in their much lower extravasation rate into the extravascular extracellular space. This characteristic enables a more precise determination of the relative cerebral blood volume (rCBV), leading to improved visualization and quantification of tumor vascularization. In the case of glioblastoma multiform for example, this is needed because distinguishing between real tumor progression and pseudo progression (treatment-induced inflammation with BBB integrity loss) is crucial for treatment monitoring. Gahramanov *et al.*^138^ put this to test in 2013 and showed that ferumoxytol can make this distinction, being a good prognostic biomarker and, unlike gadoteridol, does not require contrast agent leakage correction. Furthermore, recent concerns about neuronal tissue deposition of Gd in patients with normal renal function^139^ warrant additional studies.

Tumor macrophage content plays a substantial role in tumor angiogenesis, progression, metastasis and response to treatment. Ferumoxytol-enhanced MRI offers the first non-invasive technique clinically available for this measurement, demonstrated for high-grade gliomas by Iv *et al.*^140^ in a pilot study in 2019, where contrast enhancement correlated with iron-containing macrophages concentration.

Ghorbani *et al.*^141^ developed two nanoprobes suitable as negative contrast agents for the molecular imaging of prostate cancer.

Driven by the global imperative to combat cancer, recent years have witnessed a profound expansion in oncology research, particularly emphasizing the vast potential of SPIONs. Unlike the traditional FDA-approved compounds, many of the new nanoformulations are groundbreaking, leading to a rich tapestry of results. The core strength of SPIONs lies in their adaptability. They can be tailored with a wide array of coating materials to improve biocompatibility, and functionalised with specific targeting molecules, ensuring pinpoint accuracy to desired regions, such as cancer cells.

In breast cancer detection, formulations currently under scrutiny include SPION coated with porphyrin and functionalized with trastuzumab^142^ or SPION nanoworms conjugated with Indocyanine Green.^145^ Important research is being conducted in regard to triple-negative breast cancer, where Wu *et al.*^144^ used hybrid iron oxide–gold NPs functionalized with the Hsp70 peptide TPP *via* a PEG4 linker in order to target tumor-specific membrane Hsp70 and significantly sensitize tumor cells against radiation therapy. Early diagnosis of hepatocellular carcinoma is crucial; a phase II clinical trial^145^ conducted by Chiang *et al.*^145^ tested a newly developed SPIO in the form of iron oxide nano-particle m-PEG-silane injection, yielding this technique as safe and efficacious. Another clinical study by Hama *et al.*^146^ was able to detect every hepatic malignancy in a 0.35T MRI machine. Regarding prostate cancer, prostate-specific membrane antigen (PSMA) has emerged as the best theranostic target,^147^ warranting multiple studies that used active targeting in the form of antibodies^148^ or even an innovative PSMA-targeting Glu-Urea-Lys scaffold.^149^ Martin *et al.*^150^ targeted claudin-3 and -4 using a non-toxic *Clostridium perfringens* enterotoxin, showing overexpression in high grade prostate cancers. The overexpression of folate receptor alpha in the majority of ovarian malignancies have led to the development of anti-folate receptor alpha SPIONs that can reliably detect ovarian tumors.^151^ Lung tumors can also be visualised by taking advantage of the overexpressed folate receptor, as Kimura *et al.*^152^ showed by using polyethylene glycol-coated and dextran-coated SPIONs. Furthermore, using a hipoxia-sensitive metronidazole moiety, Yang *et al.*^153^ was able to accurately measure hipoxia in lung tumors using *T*_1_-weighted sequences, predicting tumor development. Furthermore, ongoing clinical trials are using SPION to study the tumor infiltration of glioblastoma,^154^ to assess functionally active liver parenchyma after liver cancers^155^ or to stage bladder cancer.^156^

Nevertheless, a predominant proportion of studies leveraging the tumor-imaging potential of magnetic NPs predominantly consist of theranostic platforms. Within these platforms, the inherent imaging attributes of magnetic NPs are coupled with various therapeutic strategies, thereby synergistically contributing to the mitigation of the pathological condition. This is the case in a vast array of diseases where clinical and preclinical studies are underway and magnetic NPs show a great deal of promise: brain cancer, especially gliomas,^157–159^ breast cancer,^160,161^ prostate cancer,^162,163^ ovarian cancer,^164,165^ cervical cancer,^166–168^ bladder cancer,^169–172^ lung cancer,^173^ liver cancer^174^ and even skin cancer.^175–177^ The subject of theranostic platforms and other advances of magnetic NPs will be discussed in the following section of this paper.

In summary, SPIONs are a promising tool for tumor detection in MRI. Recent research has explored ultrafine SPIONs, which can enhance tumor delivery and distribution by capitalizing on the EPR effect. They have been valuable in liver and spleen imaging, aiding in the differentiation of lesions. While some SPION-based contrast agents have been withdrawn from the market due to challenges in distinguishing liver lesions, they remain a suitable option for patients with kidney function concerns. In brain tumor imaging, SPIONs offer advantages in determining rCBV. While oncology remains a prime focus due to its pressing global significance, the versatility and advantages of SPIONs transcend this field. Their potential applicability spans across various medical domains, broadening their potential applications.

## 5. Beyond imaging: expanding horizons of magnetic nanoparticles applications

Magnetic NPs have not only demonstrated superior results in MRI compared to traditional contrast agents but their potential appears boundless, offering manifold benefits beyond current applications. While initial research predominantly honed in on their use in MRI, it's evident that we are merely at the inception of uncovering their full spectrum of capabilities in the realm of medical imaging.

At the forefront of imaging advancements stands Magnetic Particle Imaging (MPI) – a cutting-edge, non-invasive imaging technique that capitalizes on SPION, reminiscent of those utilized in MRI. Distinguished by its unique physical principles of not presenting background noise, MPI can produce optimal image contrast similar to PET/SPECT investigations, but without the irradiation. This characteristic sets it apart from MRI and positions MPI as a superior choice for quantitative determinations.

In addition, due to their facile functionalization, magnetic NPs hold a great promise for developing formulations that can be used for multimodal imaging. Their versatility allows for their integration into various imaging techniques, like CT, PET/SPECT, ultrasound, fluoroscopy or photoacoustic imaging.^178^

Especially in the oncology field, SPION have proven to be an extremely versatile compound and have been used as part of theranostic platforms employing a variety of strategies, from drug delivery to magnetic hyperthermia and the newly discovered ferroptosis. Ferroptosis is characterized by substantial iron accumulation that leads to diminished antioxidant capacity and an uptick in lipid reactive oxygen species within cells, culminating in oxidative cell death. Advancements in the field have culminated in the introduction into clinical use of a theranostic system, the Nanotherm formulation,^179^ that have gained FDA and European Medicines Agency (EMA) approvals for cancer treatment through hyperthermia. This innovative approach is currently applied in the management of conditions such as brain, prostate, and pancreatic cancers.

Given the inherent versatility and adaptability of SPIONs, the possibilities for tailoring them to specific medical needs and therapeutic strategies are virtually limitless. By integrating coating materials, fluorophores, drugs, and other active molecules, we can precisely craft them to address individual conditions and treatments, pushing the boundaries of personalized medicine and medical imaging.

## 6. Conclusion

The multifaceted world of medical imaging is experiencing a transformative era, largely driven by the advancements in nanotechnology. As elaborated in this paper, MRI stands as an invaluable tool, particularly for soft tissue analysis, due to its non-invasiveness, absence of ionizing radiation, and superior spatial resolution. Gadolinium-based NPs, which have historically been at the forefront of MRI contrast agents, have paved the way for a clearer understanding of various medical conditions. However, emerging evidence of their potential toxicity and limitations highlights the pressing need for alternative contrast agents.

Consequently, the focus has notably shifted to SPIONs due to their safety profile and customizable nature. Their ability to alter both *T*_1_ and *T*_2_ relaxation times, combined with their superparamagnetic capabilities, sets them apart. What distinguishes SPIONs from their therapeutic contemporaries is their intrinsic malleability. Their structural and functional properties can be meticulously tailored to incorporate a variety of coatings, enhancing their biocompatibility with the human system. Furthermore, by functionalizing them with specific targeting molecules, we are effectively crafting microscopic guided missiles, directing them to their intended destinations.

The versatility of SPIONs is evident from their expanding role in clinical settings. Whether it's characterizing liver lesions, tracking macrophages in inflammation and infection processes, or assessing atherosclerotic plaque stability, SPIONs have demonstrated exceptional promise. In the fight against cancer, their adaptability to be tailored with specific targeting molecules ensures that they're not just limited to imaging but can also play a pivotal role in theranostic platforms, combining diagnostic and therapeutic applications.

However, as with all advancements, challenges persist. While certain SPION formulations face drawbacks like inadequate renal clearance or potential side effects from iron overload, ongoing research and clinical trials are working relentlessly to circumvent these challenges and harness their full potential.

To encapsulate, the horizon of medical imaging, especially MRI, is vast and continually expanding. In this evolving landscape, departing from the confines of traditional FDA-approved compounds, the aforementioned avant-garde nanoformulations are establishing new frontiers in diagnostics and therapeutics. Our endeavor remains to continually innovate and optimize, ensuring the best patient outcomes while minimizing risks. The journey of exploration in the realm of nanotechnology and medical imaging is vast and ongoing, and the forthcoming chapters promise further exciting revelations and solutions.

## Conflicts of interest

There are no conflicts of interest to declare.

## Supplementary Material
